# Challenges and Opportunities for Tribal Waters: Addressing Disparities in Safe Public Drinking Water on the Crow Reservation in Montana, USA

**DOI:** 10.3390/ijerph15040567

**Published:** 2018-03-21

**Authors:** John T. Doyle, Larry Kindness, James Realbird, Margaret J. Eggers, Anne K. Camper

**Affiliations:** 1 Apsaalooke Water and Wastewater Authority, P.O. Box 126, Crow Agency, MT 59022, USA; doylej@lbhc.edu (J.T.D.); kindnesslarry@yahoo.com (L.K.); horsehand@q.com (J.R.); 2 Crow Environmental Health Steering Committee, Little Big Horn College, Crow Agency, MT 59022, USA; acamper@montana.edu; 3 Crow Water Quality Project, Little Big Horn College, P.O. Box 370, Crow Agency, MT 59022, USA; 4 Crow Tribe of Indians, P.O. Box 159, Crow Agency, MT 59022, USA; 5 Center for Biofilm Engineering, Montana State University, P.O. Box 173980, Bozeman, MT 59717, USA; 6 Department of Microbiology & Immunology, Montana State University, P.O. Box 173520, Bozeman, MT 59717, USA; 7 College of Engineering, Montana State University, P.O. Box 173820, Bozeman, MT 59717, USA

**Keywords:** drinking water, municipal water, water infrastructure, water treatment, health disparities, environmental justice, environmental health, Native American, Indian law, community-engaged research, CBPR

## Abstract

Disparities in access to safe public drinking water are increasingly being recognized as contributing to health disparities and environmental injustice for vulnerable communities in the United States. As the Co-Directors of the Apsaálooke Water and Wastewater Authority (AWWWA) for the Crow Tribe, with our academic partners, we present here the multiple and complex challenges we have addressed in improving and maintaining tribal water and wastewater infrastructure, including the identification of diverse funding sources for infrastructure construction, the need for many kinds of specialized expertise and long-term stability of project personnel, ratepayer difficulty in paying for services, an ongoing legacy of inadequate infrastructure planning, and lack of water quality research capacity. As a tribal entity, the AWWWA faces additional challenges, including the complex jurisdictional issues affecting all phases of our work, lack of authority to create water districts, and additional legal and regulatory gaps—especially with regards to environmental protection. Despite these obstacles, the AWWWA and Crow Tribe have successfully upgraded much of the local water and wastewater infrastructure. We find that ensuring safe public drinking water for tribal and other disadvantaged U.S. communities will require comprehensive, community-engaged approaches across a broad range of stakeholders to successfully address these complex legal, regulatory, policy, community capacity, and financial challenges.

## 1. Introduction

Disparities in access to safe drinking water are increasingly being recognized as contributing to health disparities and environmental injustice for tribal and other vulnerable and rural populations in the United States [1,2,3,4,5,6,7,8]. Poor quality or unsafe municipal water, denial of access to municipal water, contaminated home well water, and even lack of indoor plumbing are all contributing factors [9,10,11,12,13,14,15,16,17,18,19,20,21,22,23,24,25,26,27,28,29,30,31,32,33,34,35,36,37,38]. Several authors have identified a need to understand factors contributing to disparities associated with water infrastructure [5,7], motivating us to share lessons we have learned in addressing water infrastructure challenges in our tribal community. 

### The Crow Reservation

The Apsaálooke (Crow) people live in south-central Montana, on a reservation centered in the tribe’s original homelands. The tribe has approximately 11,000 members, about 7900 of whom live on the Reservation [2,39]. Public water and wastewater service is available for residents of the larger towns, compromising about half the population [15]. The principal Reservation towns of Crow Agency, Lodge Grass, Wyola, and Pryor are 80–95% Native American [40]. Average per capita income across the Crow Reservation ranges from $7354 to $8130, depending on the tribal community—a mere 33% of the Montana average and 30% of the 2010 national average [2,41]. The governmental headquarters of the Crow Tribe—as well as the Indian Health Service Hospital (IHS); Bureau of Indian Affairs (BIA) offices; our Tribal College, Little Big Horn College (LBHC); and other services—are all in Crow Agency, the most populous Reservation town with an estimated 2038 residents as of 2016 [40]. 

The municipal water treatment plant in Crow Agency draws surface water from the Little Bighorn River. As Crow Tribal members, we have always lived along the Little Bighorn River; we spent our childhoods playing, swimming, fishing, hunting and berry picking along the river. Our families always drew water directly from the river for both household and ceremonial consumption, and some of those practices continue today. Given our close ties to the river, we observed and remember that water quality began visibly deteriorating in the late 1970s, with the intensification of both ranching and farming, and a growing population. Our reports of evident water quality problems to the federal authorities, including leakage from municipal sewage lines directly into the river, went unresolved. We realized that the aging municipal water and wastewater infrastructure was deteriorating and inadequate to serve the growing population, and that we had to address these issues ourselves. Several of us formed the Apsaálooke Water and Wastewater Authority (AWWWA), volunteering to take on the responsibility for tribal water and wastewater infrastructure. 

In 2005, a group of tribal stakeholders and a Little Big Horn College (LBHC) faculty member conducted a week-long community wide environmental health assessment, with technical support from several federal volunteers [42,43], and concluded that water contamination was the most serious health threat affecting the greatest number of tribal members. Failing water and wastewater infrastructure was a contributing factor. We recognized that as Crow Tribal members, we needed to provide good water for our people. As AWWWA directors, we joined forces with this group, and together we and our colleagues initiated the creation of the Crow Environmental Health Steering Committee (CEHSC) [44,45] to reduce health risks and health disparities from unsafe drinking water and contaminated rivers. 

As the AWWWA and the CEHSC, we have been working with our academic partners since 2004 to improve both drinking water and river water quality on our Reservation in south-central Montana [2,44,45,46,47,48,49,50,51,52,53,54,55,56,57,58,59,60,61,62,63,64,65,66]. Community based participatory research involving a network of partners [67,68] has helped us get the data and funding needed to improve water and wastewater infrastructure in Crow Agency, our largest community [53,54]. We have been able to fund and conduct research documenting serious microbial contamination of our rivers [46,47,61]; a water treatment system in Crow Agency unable to remove all parasites (*Cryptosporidium* oocysts) during spring runoff [46,47]; widespread metals, nitrate, and microbial contamination of home well water [2,16,50,52,57,58,59,60,62,64,65]; and threats to our water supplies and hence community health from climate change [49,55,56].

In this paper, we and our CEHSC colleagues (Eggers and Camper) describe difficulties encountered in repairing and replacing Crow Tribal public water and wastewater infrastructure, and some of the strategies that have been helpful in overcoming the hurdles. Some of these challenges are shared by many vulnerable rural communities [1,5,6,7,8,10,11,17,19,21,38]; others stem from laws and regulations (or lack thereof) unique to Native American communities. 

## 2. Water and Wastewater Infrastructure Improvements

The water and wastewater infrastructure in Crow Agency was built more than 100 years ago, to serve a population less than half of what it is today. No major upgrades of the distribution system nor the sewage lagoon had been undertaken since. Over the past 10 years, the AWWWA—with support from our partners—has secured almost 50 federal, state, county and tribal grants and several loans totaling more than $20 million to date. With this funding, (1) the inadequate, failing sewage lagoon has been replaced with a modern one; (2) 75% of old wastewater lines have been replaced; (3) new, upgraded water and wastewater connections to local schools have been installed; (4) about 50% of water lines have been replaced; (5) a “water salesman” was installed to sell town water at minimal cost to any community member; and (6) 19 broken fire hydrants were replaced. This work continues, with the relocation of the wastewater lift station (out of the floodplain) being one of our current priorities. In the process, we have learned that many kinds of expertise are essential to this work, and that the challenges are exacerbated by legal and jurisdictional issues unique to Reservation communities [53,54]. 

### 2.1. The Challenges of Improving Water and Wastewater Infrastructure

Rural communities, especially those with limited economic opportunities, face multiple challenges in funding, building and successfully operating water and wastewater infrastructure [1,5,6,7,8,10,11,17,19,21,38]. In our experience, tribal communities face these as well as additional unique challenges. 

First, and not unique to tribes, this work takes community-wide self-determination and initiative. Learning and following the tenets of community-based participatory research (CBPR) [67,68] has been both helpful and essential. Operating according to CBPR principles has given our community ownership of the work and provided the broad support necessary to carry out this long-term effort. CBPR has supplied an effective framework to collaborate with academic partners on the needed water quality research [44,45].

Project leadership must make a long-term commitment to this work. Renovating municipal water and wastewater infrastructure is an enormous, complicated, and lengthy job, requiring substantial experience and institutional memory. If the staff turned over every two or four years with tribal elections (as often happens), the work would be impossible. The Crow Tribe created the Apsaalooke Water and Wastewater Authority (AWWWA) as an independent entity, which has allowed continuity.

Water quality data are essential for understanding health threats from contaminated water sources, and for raising the funds to mitigate them through water and wastewater infrastructure upgrades. Our decades of observations of river water quality deterioration lacked the scientific credibility necessary to support grant proposals. Consequently, we partnered with researchers at Montana State University Bozeman (MSU) and at LBHC to secure research funding, obtain access to lab facilities and conduct the requisite water quality research. The surface and groundwater data subsequently obtained, elucidating microbial and mineral contamination, have supported successful infrastructure grant proposals and informed the community of health risks from water sources. We—and tribal college science majors—have gained research experience in the process, building our tribe’s capacity to conduct research with academic partners. We are committed to sharing our results in our own communities, as well as through webinars and conference presentations such as the National Congress of American Indians’ Tribal Leader Scholars Forum and through journal publications [2,16,44,45,46,47,48,49,50,51,52,53,54,55,56,57,58,59,60,61,62,63,64,65,66]. 

In addition to research data, access to grant writing expertise and knowledge of the diverse funding sources for water and wastewater infrastructure are essential. Learning where to seek funding has been one of the more difficult tasks. Partnering with an excellent grant writer has helped [69], but we have also learned about diverse funding sources by actively seeking out opportunities through our networks, and by listening to and reading the news. Once funding is secured, federal and state grant administration expertise is necessary. AWWWA Director Doyle, researcher Eggers and the LBHC Grants Manager all completed professional short-course training in federal grants administration, which was worthwhile [70]. An additional issue is that many funding sources operate on a reimbursement basis, which requires that the tribe or water authority has adequate liquidity to be able to front the funds.

As AWWWA Directors, we must have sufficient engineering knowledge to make informed decisions about many aspects of water and wastewater infrastructure, as well as a trusted engineering firm with whom to partner [53,54]. We hear time and again of tribal and other minority communities purchasing water and wastewater systems, without being fully aware of or planning for the operation and maintenance costs, only to discover that they cannot afford to operate the new systems they have acquired. If engineering firms were required to estimate operation and maintenance costs when they propose new treatment systems, tribes and other communities would be in a better position to make informed decisions about major infrastructure purchases [71].

### 2.2. Tribes Face Additional Complexities and Hurdles in Addressing Water Infrastructure Needs

Ongoing access to legal counsel is essential, especially for tribes. Jurisdictional issues for infrastructure work on tribal lands are multiple, complex, challenging, and time consuming to resolve. Policy and regulatory challenges stem particularly from overlapping, conflicting and/or gaps in tribal/federal/state/county jurisdiction on Reservations, further complicated by intersecting responsibilities of the tribe, county, Indian Health Service, Bureau of Indian Affairs (BIA), and the Environmental Protection Agency. 

#### 2.2.1. Obtaining Rights of Way

Whenever renovations are made to water distribution systems or wastewater collection and treatment systems, new rights of way are often needed. On Reservations, obtaining rights of way is complicated by tribal and individual trust lands administered by the BIA, in addition to the usual federal/state/county/railroad/private mix of land ownership. As the BIA explains [72]:

A federal Indian reservation is an area of land reserved for a tribe or tribes under treaty or other agreement with the United States, executive order, or federal statute or administrative action as permanent tribal homelands, and where the federal government holds title to the land in trust on behalf of the tribe.

This goes back to an 1823 U.S. Supreme Court decision, Johnson vs. McIntosh, 21 U.S. (8 Wheat.) 543 (1823), page 64, supra, which ruled that the tribal government has a right of occupancy—not ownership of fee title—to their Reservation, which is held “in trust” for them by the federal government [73]. When the Dawes Act was passed in 1887, individual parcels of the Crow Reservation were allotted to tribal members, who then had a right of occupancy and use of their “allotment”, although the land was still in Trust [74]. After 21 years, they could apply to remove their allotment land from Trust, convert it to fee simple ownership, pay real estate taxes on the land and sell it if they wished to do so. As a result, all these types of land ownership exist today (tribal trust, individual trust, fee simple) in a checkerboard pattern across the Reservation. Each type of land ownership requires a different process to obtain a right of way (ROW) to lay water or wastewater lines. For instance, while it is possible to get an ROW either parallel to or across a state road, federal roads only allow ROWs across (but not parallel to) the road. Rights of way across Trust lands, whether ‘occupied’ by the tribal government or by an individual, require permission from the occupants first, and then from the BIA. The BIA requires an environmental assessment be done before an ROW will be granted, and this is a lengthy process. It is essential to understand all the different ROW requirements for each type of land ownership when planning a water/wastewater infrastructure project, so that the water lines are planned for routes where ROW can be obtained, and the project budget covers all the anticipated ROW expenditures.

#### 2.2.2. Fractionation of Land Ownership

ROWs across Trust lands are further complicated by ‘fractionation’ of land ownership. Individual allotted lands have now been handed down to multiple heirs as tenants in common, through multiple generations over the past 100 years, resulting in highly fractionated lands. One piece of trust land can have hundreds of people sharing a small parcel with undivided interests [75,76,77,78]. In a thorough analysis of “Allotment, fractionation, and the Indian land tenure problem”, Shoemaker writes [75]:

The personal and political effects of fractionation are critical for both tribal governments and their citizens. Fractionation prevents efficient use of property, impedes individual and community economic development, and fundamentally bars realization of successful tribal self-determination and self-governance.

For the AWWWA, fractionation has created complex, difficult, and expensive issues with obtaining the necessary rights of way to replace water and wastewater distribution lines. Although the Crow Tribal government does have the authority to override the BIA ROW process and any objections by the occupants of the Trust land in question, neither the tribal government nor the AWWWA wished to resort to this harsh procedure. 

#### 2.2.3. Tribal Lack of Eminent Domain Authority

Obtaining ROWs across lands held in fee simple presents other challenges, if landowners are not willing to grant an ROW at market value. Whereas counties can resort to eminent domain procedures, tribal governments do not own their land in fee simple [72,73], and therefore do not have clear eminent domain authority [79]. In our case, the county was blocked from using its eminent domain authority to help the tribe. The state’s authority became our only recourse. While we were eventually able to obtain the necessary rights of way with the help of an eminent domain attorney, it became a lengthy, sometimes contentious and very expensive process.

#### 2.2.4. Conflicting Regulations Concerning Tribal Preference in Hiring and in Awarding Contracts

Tribes have the legal right to adopt a Tribal Employment Rights Ordinance (TERO), called Workforce Protection, to boost tribal employment when the source of funding is tribal income or federal Public Law 93–638 contracts [80]. However, TERO cannot be applied to the award of contracts or hiring for jobs which are funded by or through the State of Montana, such as Community Development Block Grants. Many water infrastructure projects are so costly that multiple sources of funding—with different requirements for the application of Indian preference and payment of TERO fees—must be combined to be able to finance a single project. This conflict has the potential to derail projects altogether [54]. 

#### 2.2.5. Sovereign Immunity

The award of grants from the State of Montana comes with an additional requirement: partial waiver of sovereign immunity. Under Montana Code Annotated 90-6-209, the Coal Board of the State of Montana cannot award a grant to a tribe unless [81]:

The governing body of the tribe has agreed:

(1) To waive its immunity from suit on any issue specifically arising from the transaction of a grant obtained under this part; and
(2) To the adjudication of any dispute arising out of the grant transaction in the district court of the first judicial district of the State of Montana…

Each state agency has similar, but slightly different requirements for waivers of sovereign immunity, so each state grant requires negotiation with the tribe as to the precise terms. Once the exact wording has been agreed upon, the Crow Tribal Chairman and the Crow Tribal Legislature must agree to a limited waiver of sovereignty for the grant transaction, by passing a Joint Action Resolution [82], another process that can be difficult and time consuming.

#### 2.2.6. Dispute Resolution

These legal and regulatory hurdles and inconsistencies can be challenging to resolve to all parties’ satisfaction. Planning in advance for dispute resolution is necessary. Funding agencies want disputes to go to federal District Court (e.g., [81]), while the tribe wants them to go to Tribal Court, and then Federal Court if need be. The procedure for settling disputes must be negotiated and included in contracts. Arbitration can help [54].

#### 2.2.7. Lack of Planning

The historic and ongoing lack of planning impacts infrastructure construction and operation. Housing, even federally funded housing, is still being built without adequate infrastructure planning. For instance, housing has been built where there is insufficient water pressure in the distribution system to accommodate more homes, despite the expressed concerns of the AWWWA. The lift station serving the south end of Crow Agency was built in the flood zone; it was so damaged in the 2011 flood that wastewater service was shut down for a couple weeks, including to the Indian Health Service hospital. Spring floods appear to be increasing in severity and frequency [56], but the capacity and funding to plan for and adapt to worse floods and other severe weather events are lacking. The AWWWA is left to solve physical and fiscal problems which could have been avoided by adequate planning [53,54].

#### 2.2.8. Legacy of Inadequate Environmental Enforcement

A long and continuing history of poor environmental enforcement also contributes to the expenses of building, maintaining and operating water infrastructure. Below the land surface, there is a dumping ground resulting from a century of neglect and abandonment. When distribution lines are installed or replaced, cleaning up unmapped, unanticipated underground hazards significantly delay schedules and increase costs [54]. For instance, there is an abandoned steam-generating power plant in Crow, which used to heat some of the federal buildings. The underground asbestos-wrapped steel lines for steam delivery were never removed, and no complete map of the steam distribution system exists. When the AWWWA excavated for water and wastewater lines, the excavators would run into these abandoned steam lines, which triggered a requirement to develop an asbestos mitigation plan. Such plans had to be approved by multiple departments of the BIA and then implemented—another costly and time-consuming process. 

The river source water for the Crow Agency water treatment plant has severe microbial contamination in the spring, to which non-point source pollution from agriculture [61], failing home septic systems and straight piping from homes (i.e., completely untreated wastewater) all contribute. There is no agency taking responsibility for permitting and inspecting home septic systems on the Reservation. The water treatment plant’s technology cannot effectively handle the springtime spike in pathogen load, for instance, from *Cryptosporidium* oocysts [46,47].

#### 2.2.9. Lack of Tribal Authority to Create Water Districts

Tribal budgets to operate and maintain water and wastewater systems cannot be supported through the mechanisms available to non-tribal communities, as tribes lack the legal authority to create a water district. Off reservation, communities form water districts, vote on members to run the district and tax residents to support water and wastewater systems. However, since this authority comes from state law, it is not available to Reservation communities. Without the ability to form a water district, tribes have less ownership, and lose the ability to raise funds through taxation and to guarantee the construction loans as required by some funding agencies. Even if major infrastructure can be upgraded or replaced through grants, inadequate operation and maintenance budgets mean dedicated operators are underpaid. In Crow, due to budget constraints, operators routinely take on major repair jobs that most utilities would contract out. The water and wastewater plants are severely under staffed and some maintenance and repair needs beyond the expertise of the operators (such as damage to computerized systems) simply go unaddressed.

#### 2.2.10. Lack of Operator Certification Requirements

The requirement for water treatment plant operators to be certified has long been a state function [83], without any comparable federal requirement for tribal lands (which are not under State jurisdiction). Hence, adequate tribal funding has not been provided for operators to obtain training for certification, and even operators who have years of valuable on the job water and wastewater experience have no easy path to obtain certification. This leaves tribal administrators with no clear standard to assess the qualifications of applicants for these positions. This landscape is starting to change as the regional EPA office emphasizes the need for certified operators, but it remains difficult for tribal operators to acquire all the necessary training and skills anywhere within driving distance.

## 3. Conclusions

Building, operating, and maintaining effective water and wastewater infrastructure is difficult for many vulnerable communities, especially those with limited financial, technical, and administrative resources. Inability to meet all these challenges can result in communities not receiving safe public drinking water, and hence contributes to health disparities. Some of the issues described above are not unique to tribes, e.g., the need for long term stability of project personnel; access to many kinds of specialized expertise; ratepayer difficulty in paying for services; lack of knowledge of funding sources for water infrastructure; and lack of research capacity to assess and document water quality issues. As a tribal entity, the AWWWA faced and continues to address additional challenges, including the complex jurisdictional issues affecting many aspects of fundraising, design, contracting and construction; lack of authority to create water districts; other legal and regulatory gaps—especially with regards to environmental enforcement; a legacy of inadequate infrastructure planning and the potential for repeated personnel turnover with the tribal election cycle. Despite these obstacles, the AWWWA and Crow Tribe have been successful in replacing and upgrading water and wastewater infrastructure for the Reservation’s largest community. The AWWWA’s work is ongoing, as decades of deferred maintenance, population growth and worsening source water quality have left every Reservation community with unmet water infrastructure repair and replacement needs. Doyle, Eggers, Camper, and other members of the CEHSC continue to work on other water quality issues on the Reservation, in collaboration with LBHC and MSU. 

Ensuring safe public drinking water for tribal and other disadvantaged communities throughout the United States will require comprehensive, community-engaged approaches across a broad range of stakeholders to successfully address these complex legal, regulatory, policy, financial and capacity challenges.
